# Using network analysis to explore the association between eating disorders symptoms and aggressiveness in *Bulimia nervosa*

**DOI:** 10.3389/fpsyt.2022.907620

**Published:** 2022-08-24

**Authors:** Giammarco Cascino, Francesca Marciello, Giulia D’Agostino, Rita Toricco, Eugenia Barone, Alessio Maria Monteleone

**Affiliations:** ^1^Section of Neurosciences, Department of Medicine, Surgery and Dentistry “Scuola Medica Salernitana,” University of Salerno, Salerno, Italy; ^2^Department of Psychiatry, University of Campania “Luigi Vanvitelli,” Naples, Italy

**Keywords:** eating disorders, bulimia nervosa, aggressiveness, hostility, network analysis

## Abstract

Aggressive behaviors have been reported to be more frequent in people with eating disorders (ED), especially bulimia nervosa (BN). Network Analysis (NA) is particularly useful or examining the interactions among symptoms of comorbid conditions through the identification of “bridge symptoms,” defined as those symptoms playing a key role in the connection between two syndromic clusters. The aim of the present study was to investigate the association of ED core symptoms and ED-related psychopathology with aggressiveness in a clinical sample of women with BN through NA. Two hundred and seventy-nine women with BN completed the Eating Disorder Inventory-2 and the Buss-Durkee Hostility Inventory. A NA was conducted, including ED symptoms and aggressiveness measures. The bridge function was implied to identify symptoms bridging ED symptoms and aggressiveness. The most connected nodes among communities were asceticism and impulsivity from ED-related psychopathology, drive for thinness from ED-core psychopathology and guilt and suspicion from aggressiveness domain. In particular, drive for thinness connected ED-core community to verbal hostility, while impulsivity connected ED-related symptoms to guilt and suspicion of aggressiveness community. In conclusion the present study showed that in people with BN guilt is the specific negative emotion of the hostile dimensions that may be bidirectionally associated with ED symptoms.

## Introduction

Aggressiveness is defined as the intention to harm someone (1, 2). However, a distinction should be made between instances where harming others is secondary to the main goal of obtaining an advantage for themselves and other forms of aggressive behaviors in which the intention to harm someone is primary and is raised by emotionally driven lack of impulses control (3). The affective-driven form of aggressive behaviors is conceived as maladaptive and source of conflict between the subject and his family and/or peers (4). Aggressive behaviors have been reported to be more frequent in people with eating disorders (EDs), especially bulimia nervosa (BN), than in non-clinical populations (5–7). They were associated with the severity of bulimic symptoms (5, 8), self-harming behaviors (8, 9), comorbidity (10), and poorer treatment outcome (5, 11).

The association between aggressiveness and ED symptoms has been investigated considering the mediation role of several factors. First, people with ED and comorbid major depression disorder were characterized by irritable mood and showed anger attacks and angry outbursts with a prevalence approximately double than depressed patients without ED (10). A few studies have assessed anger and personality concomitantly in people with BN and, even if they did not reveal replicable patterns of association between anger and personality characteristics, a full mediation of anger between cooperativeness and binge-eating and impulsiveness has been found, supporting a relationship between anger feelings and interpersonal difficulties (12, 13). Finally, impulsive behaviors, such as self-injurious behavior and drug or alcohol abuse, have consistently been found associated with anger and eating disorder symptoms (14, 15). Because of wide differences across studies in methods and sample composition, the relationships between aggressiveness and ED psychopathology are not conclusive and need to be further investigated.

The application of network analysis (NA) in the study of psychopathology has become more common in the recent years. Indeed, the NA approach is believed to be a particularly useful tool for examining the interactions among symptoms of comorbid conditions (16). Networks are composed of nodes, representing the assessed symptoms of a psychiatric syndrome, and edges, which are the connections among nodes. A further use of NA is the identification of “bridge symptoms,” defined as those symptoms playing a key role in the connection between two syndromic clusters, namely, groups of symptoms that are very closely related (17). Therefore, it is thought that the nodes with the highest bridge centrality are spreading the activation of symptoms from one disorder to another and promote psychiatric comorbidity (18). A recent systematic review (19) showed that, in addition to ED-specific symptoms (i.e., overvaluation of shape and weight), anxious and depressive symptoms are central network nodes across ages and diagnoses in EDs. This review also included studies that examined bridge centrality in ED networks (19) and identified low self-esteem and interoceptive awareness as connecting ED-related symptoms to anxious and depressive symptoms (20–23). However, this review highlighted the need for future studies to include in the network nodes related to general psychopathology in addition to those specifically related to ED symptoms. To our knowledge, no study so far has applied the NA and the bridge function to examine the relationships between aggressive behaviors and ED symptoms.

Therefore, the aim of the present study was to investigate the association of ED core symptoms and ED-related psychopathology with aggressiveness in a clinical sample of women with BN. First, to identify the most central symptoms of each psychopathological domain, two separate networks were estimated: one for ED psychopathology and one for the aggressiveness dimension. Second, a network including both ED psychopathology and aggressiveness related features was estimated to identify bridge nodes between the ED symptoms and aggressiveness clusters.

## Materials and methods

### Assessment

Consecutive patients attending the Eating Disorder Centre of the Department of Psychiatry at the University of Campania “Luigi Vanvitelli” were included in the study if they met the following criteria: (a) female sex; (b) age ≥ 18; (c) current diagnosis of BN according to the fifth edition of Diagnostic and Statistical Manual of Mental Disorders (DSM-5) criteria and (d) willingness to sign a written informed consent. Trained psychiatrists made diagnostic assessment by means of the Structured Clinical Interview or DSM-5 Disorders-Research Version (24) and collected sociodemographic and clinical data. The Italian version of Eating Disorder Inventory-2 (EDI-2) (25) and Buss-Durkee Hostility Inventory (BDHI) (26) were completed at admission before starting specific treatment programs. The study was approved by the Institutional Board of the “Department of Mental Health, Physical Health, and Preventive Medicine” of the University of Campania “Luigi Vanvitelli,” Naples, Italy.

The EDI-2 is a 92 items self-report questionnaire, which provides 3 subscales measuring the ED core psychopathology (i.e., drive for thinness, body dissatisfaction, and bulimia) and 8 subscales measuring ED-related psychopathology: ineffectiveness, maturity fear, social insecurity, perfectionism, interpersonal distrust, impulsivity, interoceptive awareness, and asceticism. Cronbach’s α ranged from 0.90 (drive for thinness) to 0.71 (maturity fear).

The BDHI consists of 75 dichotomous items specifically developed to explore different subtypes of hostility and guilt (27). Heightened scores on the BDHI reflect greater hostility. This questionnaire includes the following subscales: assault, indirect hostility, irritability, negativism, resentment, suspicion, verbal hostility, guilt. Cronbach’s α for the BDHI total score was 0.84.

### Network analysis

In the present study, partial correlation networks were estimated in which nodes represent observed variables and edges represent partial correlation coefficients between two variables after conditioning on all other variables in the dataset (28). Network associations do not imply any directionality, which means the lack of any causal inference or direction in the association. The thickness of an edge graphically represents the magnitude of the association. NA was performed through R, version 3.4.4, using qgraph package (29) that utilizes the package glasso in combination with Extended Bayesian Information Criterion (EBIC) model selection (30). The “least absolute shrinkage and selection operator” (LASSO) is a form of regularization, which applies a penalty by limiting the total sum of absolute parameter values leading many edges to zero and dropping them out of the model, returning a sparse and more interpretable network (31). The LASSO utilizes a tuning parameter to control the degree to which regularization is applied, i.e., EBIC, which was set to 0.5 in the analyses. Individuals with missing data (*n* = 21) were excluded from the analysis.

A typical way of assessing the importance of nodes in the network is to compute centrality indices of the network structure (32). The most informative is the node strength, that is the sum of the absolute edge-weights between a node and all other nodes to which it is connected in the network.

Following the Epskamp et al. recommendations (33) to assess the accuracy of the edge-weights and strength centrality index, we performed non-parametric bootstrap (nboots = 2,500) and obtained bootstrapped confidence intervals (CI) showing the accuracy of edge-weight estimates. Then we proceeded to the calculation of the correlation stability coefficient, which is the maximum proportion of population that can be dropped so that the correlation between the recalculated indices of the obtained networks and those of the original network is at least 0.7. Analyses of network accuracy were performed using the bootnet package (34).

To identify the bridge symptoms, which play a primary role in connecting two or more symptom clusters (17), we estimate the bridge strength centrality index, which is the absolute sum of all edges that connect a node to all nodes that are not part of the same community (35). Thus, a high bridge expected value indicates that the node is strongly connected to other nodes included in other communities. The *bridge* function from *networktools* package was used (36). We applied *a priori* definition of communities that can be defined as clusters of symptoms. Three communities were defined: the first referred to ED core psychopathology and included EDI-2 drive for thinness, body dissatisfaction and bulimia sub-scores; the second was related to ED-related psychopathology and was composed by the other sub-scores of the EDI-2; the third was related to aggressiveness and included all sub-scores from the BDHI. First, all communities were considered for the estimate of bridge strength centrality, then communities to be included in the analysis were specified to disentangle the bridge symptoms connecting each community to the other two.

## Results

Three hundred women met the inclusions criteria. Two hundred-seventy-nine completed the self-report questionnaires. Demographic and clinical characteristics of the study population are reported in Table 1. Sixty-nine patients were taking antidepressant medications, namely SSRI.

**TABLE 1:** Demographic and clinical characteristics of the study population.

Age, years	26.1 ± 5.5
Body Mass Index, kg/m^2^	21.5 ± 2.6
Illness duration, years	5.3 ± 3.9
Comorbidity, yes	87 (30)
Depression, yes	43 (14.5)
Anxiety, yes	38 (13)
DOC, yes	6 (2)
Previous AN, yes	100 (34)
Drive for thinness	14.7 ± 5.9
Bulimia	10.4 ± 6.0
Body dissatisfaction	15.5 ± 7.5
Ineffectiveness	11.6 ± 6.9
Perfectionism	5.4 ± 4.3
Interpersonal distrust	6.4 ± 4.6
Interoceptive awareness	11.5 ± 6.9
Maturity fear	7.9 ± 5.6
Asceticism	7.4 ± 4.4
Impulsivity	8.2 ± 6.7
Social insecurity	8.3 ± 4.8
Assault	51.4 ± 11.5
Indirect hostility	54.8 ± 11.0
Irritability	53.5 ± 8.3
Negativism	50.8 ± 10.7
Resentment	61.4 ± 12.3
Suspicion	56.1 ± 12.1
Verbal hostility	49.5 ± 10.7
Guilt	60.4 ± 9.2

**FIGURE 1:**
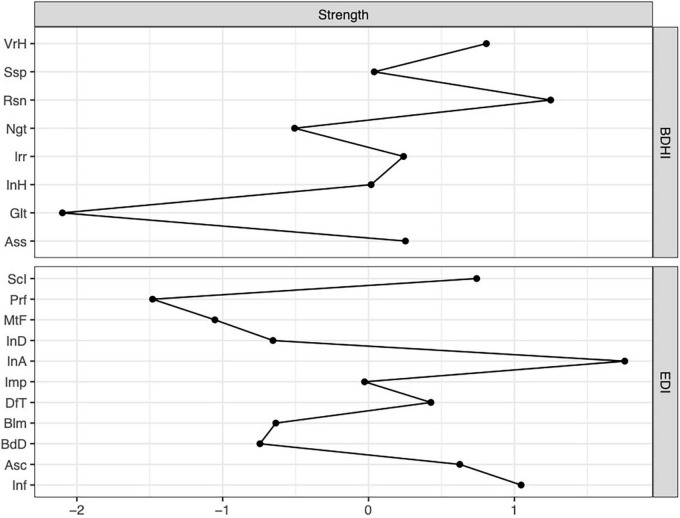
Plot of strength centrality index of the separate network for Eating Disorder Inventory-2 (EDI) and Buss-Durkee Hostility Inventory (BDHI). VrH, Verbal hostility; Ssp, Suspicion; Rsn, Resentments; Ngt, Negativism; Irr, Irritability; InH, Indirect hostility; Glt, Guilt; Ass, Assault; ScI, Social Insecurity; Prf, Perfectionism; MtF, Maturity Fear; InD, Interpersonal distrust; InA, Interoceptive awareness; Imp, Impulsivity; DfT, Drive for Thinness; Blm, Bulimia; BdD, Body Dissatisfaction; Asc, Asceticism; Inf, Ineffectiveness.

In the network including ED-core and related psychopathology the node with the highest strength centrality was interoceptive awareness (*M* = 1.27), followed by ineffectiveness (*M* = 1.07), while in the network composed by BDHI measures the node with the highest strength centrality was resentment (*M* = 1.05), followed by verbal hostility (*M* = 0.99) (Figure 1).

**FIGURE 2:**
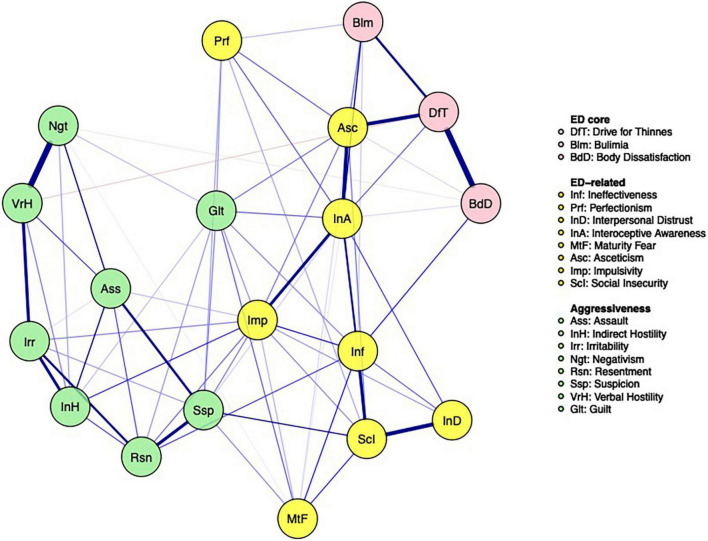
Estimated partial correlation network of the study population.

**FIGURE 3:**
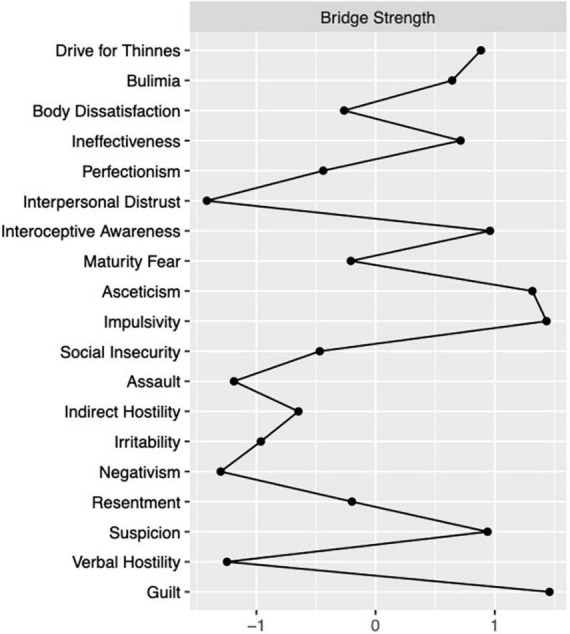
Plot of bridge strength centrality index considering all symptoms communities.

The partial correlation network including both aggressiveness variables and ED-core and related psychopathology is reported in Figure 2. When considering all the communities in the estimate of bridge strength centrality, the most connected nodes among communities were asceticism and impulsivity from ED-related psychopathology, drive for thinness from ED-core psychopathology and guilt and suspicion from the hostility domain (Figure 3). When considering the ED-core and the ED-related communities, the nodes with the highest bridge strength were the ED-core psychopathological dimensions drive for thinness and bulimia (*M* = 0.34 and 0.33, respectively) and the ED-related psychopathological dimensions asceticism and interoceptive awareness (*M* = 0.36 and 0.26, respectively). When considering the ED-core and the hostility communities, the nodes with the highest bridge strength were drive for thinness and verbal hostility (*M* = 0.03, both) connected to each other by a negative edge. Finally, when considering the ED-related psychopathology and the hostility communities, the nodes with the highest bridge strength were impulsivity (*M* = 0.45) from ED-related psychopathology and guilt and suspicion (*M* = 0.46 and 0.38, respectively) from hostility community. Plots of bridge strength centrality are reported in the supplementary materials. Centrality stability coefficient for strength of the network of ED-psychopathology was 0.52, while that of the network of hostility symptoms was 0.44. Centrality stability coefficient of the network including both ED and hostility symptoms was 0.43 for bridge strength.

## Discussion

The current study investigated the psychopathology related to ED-specific symptoms and to the aggressiveness dimension in individuals with BN through NA. Also, the association between hostility and ED psychopathological features was evaluated through the bridge function analysis. The first finding of our study was that interoceptive awareness and ineffectiveness were the most central nodes in the network composed by measures of ED psychopathology, while resentment and verbal hostility were the most central nodes in the network composed by the BDHI sub-scores.

The high centrality of ineffectiveness and interoceptive awareness is consistent with previous studies exploring ED psychopathology through self-report questionnaires assessing not only ED-specific psychopathological features but also general psychopathology (19). Previous studies which included EDI-2 sub-scores in the networks merged individuals with different ED diagnoses in a unique ED group (37, 38) or were conducted in people with AN (39, 40). Only one study (41) has conducted an assessment of ED psychopathology through the EDI-2 in people with BN and found that ineffectiveness, anxiety, and depression scores and interpersonal sensitivity were the most central nodes in a network composed also by measures of general psychopathology and personality dimensions. Our findings are partially consistent and support the hypothesis that not only ED specific symptoms (e.g., bulimic symptoms measured through the corresponding EDI-2 subscale) but also more general features of psychopathology are essential in BN. The importance of ineffectiveness in individuals with BN has also been shown in a previous network study (42) which showed that this node was included in the shortest pathway between early traumatic experiences and ED-related core symptoms.

The second finding of the present study was that in the network composed by BDHI sub-scores, resentment, and verbal hostility were the nodes most connected to others. While resentment refers to jealousy and hatred of others, verbal hostility refers to expression of negative affect in both the style and content of speech (43). These variables both showed strong connection with irritability, while resentment showed also a strong connection with suspicion (i.e., the projection of hostility into others) and verbal hostility was strongly and positively associated with negativism, that is an oppositional behavior usually directed against authority. The lack of directionality of the current network edges and the cross-sectional nature of these findings do not allow to distinguish the direction of these connections: thus, a bidirectional relationship between these dimensions should be interpreted. Future studies will have to clarify if this aggressiveness profile characterizes individuals with BN and differs between the main ED diagnoses.

The partial correlation network including both aggressiveness variables and ED-core and related psychopathology with the application of the bridge strength estimates showed the importance of impulsivity, interoceptive awareness, suspicion, and guilt in the association between ED-core symptoms and aspects of hostility. Even if it is not possible to draw causal inference from the connections among variables, since network edges are undirected, the associations between these nodes may be interpreted in two ways. First, both suspicion and guilt could arise from the feeling of being unable to control body shape and weight or from conflicts due to disordered eating behaviors with feelings of being bad and having done wrong. Indeed, patients may project hostility onto others when they are not able to accomplish disordered eating behaviors because they feel to be inhibited by others. Second, suspicion and guilt may represent the negative affects that lead to eating psychopathology in individuals with BN. Indeed, people with a lifetime diagnosis of EDs showed an attentional bias to rejecting faces and a difficulty disengaging attention from these stimuli (44). This attentional bias to rejection was found to be positively correlated with adverse childhood experiences, which have been associated both with higher rates of aggressive behaviors and EDs symptoms (45, 46). Moreover, the negative interpretation bias toward social stimuli correlated with clinical symptoms (47). The tendency to act impulsively in response to negative emotions may trigger loss-of-control eating episodes (48). Indeed, negative mood has been found to precede often binge eating episodes and compensatory behaviors in patients with BN (49). Furthermore, interoceptive awareness is highly associated with the hostile dimensions. Impaired interoceptive awareness play a central role in the pointing to reduced trusting and listening of visceral signals related to body states for emotional information and behavioral guidance (50). Also, individuals with EDs show low ability to cope with distress by attending to their body signals (50). The current findings outline a possible connection between this psychopathological feature and hostile dimensions suggesting that unhealthy eating behaviors may serve to manage uncomfortable body sensations when perceiving guilt or hostile feelings.

The present study presents some limitations that need to be acknowledged. First, the use of cross-sectional data does not allow to draw causality effects. Second, it must be considered that the use of self-report questionnaires, although consistent with previous literature, may have led to the underestimation of aggressive behaviors. Then, assessments of personality traits and measures of general psychopathology, such as depression or anxiety, which are frequent in EDs (51), are lacking. Indeed, psychiatric comorbid depressive or anxiety syndromes could have affected anger expression, since people with ED and comorbid major depressive disorder exhibited outbursts of anger twice than depressed patients without ED (10). From a clinical point of view, if confirmed by future studies, the observed connections of hostility dimensions with impulsivity and interoceptive awareness may be worth of therapeutic attention.

This supports the importance to assess impulsivity to offer proper treatment for individuals with BN. Indeed, treatments that aim to manage impulsive behaviors can be particularly helpful as they act on what connects the ED psychopathology to hostility, which is known to worsen the outcome of treatments (11). For example, the dialectical behavior therapy aiming to manage impulsive behaviors (52) is a skills-based approach for teaching individuals general problem-solving skills, emotional regulation strategies, interpersonal skills, and distress tolerance (53). It could help to improve hostility behaviors, and it has been adapted and applied to the treatment of individuals with BN (54).

## Conclusion

In conclusion the present results show for the first time that in people with BN guilt is the specific negative emotion of the hostile dimensions that may be bidirectionally associated with ED symptoms, although the verbal hostility and resentment are highly central in the hostility profile of these individuals. This may point to an ambivalent profile of people with BN which needs to be further explored in future studies.

## Data availability statement

The raw data supporting the conclusions of this article will be made available by the authors, without undue reservation.

## Ethics statement

The studies involving human participants were reviewed and approved by Institutional Board of the “Department of Mental Health, Physical Health, and Preventive Medicine” of the University of Campania “Luigi Vanvitelli,” Naples, Italy. The patients/participants provided their written informed consent to participate in this study.

## Author contributions

GC and AM designed the study, wrote the protocol, and wrote the manuscript. FM, GD’A, and RT collected the data. GC and EB performed the statistical analyses. All authors contributed to the article and approved the final version of the manuscript.
